# Supplementary material for: Full integration of highly stretchable inorganic transistors and circuits within molecular-tailored elastic substrates on a large scale
Source: Nat Commun. 2024 Apr 1;15:2814. doi: 10.1038/s41467-024-47184-w (PMC10985077; doi:10.1038/s41467-024-47184-w)
Supplement: Supplementary file 1 — Supplementary Information [file 41467_2024_47184_MOESM1_ESM.pdf]

Supplementary Information

Full Integration of Highly Stretchable Inorganic Transistors and Circuits within Molecular-Tailored Elastic Substrates on a Large Scale

Seung-Han Kang[1,2,9], Jeong-Wan Jo[3,9], Jong Min Lee[1,2], Sanghee Moon[2], Seung Bum Shin[4], Su Bin Choi[5], Donghwan Byeon[1,2], Jaehyun Kim[6], Myung-Gil Kim[4], Yong-Hoon Kim[4*], Jong-Woong Kim[5,7,8*], and Sung Kyu Park[1,2*]

[1] Department of Intelligent Semiconductor Engineering, Chung-Ang University, Seoul 06974, Korea

[2] School of Electrical and Electronic Engineering, Chung-Ang University, Seoul 06974, Korea

[3] Electrical Engineering Division, Department of Engineering, University of Cambridge, 9 JJ Thomson Avenue, Cambridge CB3 0FA, UK

[4] School of Advanced Materials Science and Engineering, Sungkyunkwan University, Suwon 16419, Korea

[5] Department of Smart Fab. Technology, Sungkyunkwan University, Suwon 16419, Korea

[6] Department of Semiconductor Science, Dongguk University, Seoul 04620, Republic of Korea

[7] School of Mechanical Engineering, Sungkyunkwan University, Suwon 16419, Korea

[8] Department of Semiconductor Convergence Engineering, Sungkyunkwan University, Suwon 16419, Korea

[9] These authors contributed equally: Seung-Han Kang, Jeong-Wan Jo

*Corresponding Authors: Prof. Sung Kyu Park, Prof. Yong-Hoon Kim, and Prof. Jong-Woong Kim

Phone: 82-2-820-5347, Email: skpark@cau.ac.kr, yhkim76@skku.edu, wyjd@skku.edu,

This "Supplementary Information" includes:

Supplementary Figure 1 to 19,

Supplementary Table 1 to 3,

Supplementary Reference 1 to 2,

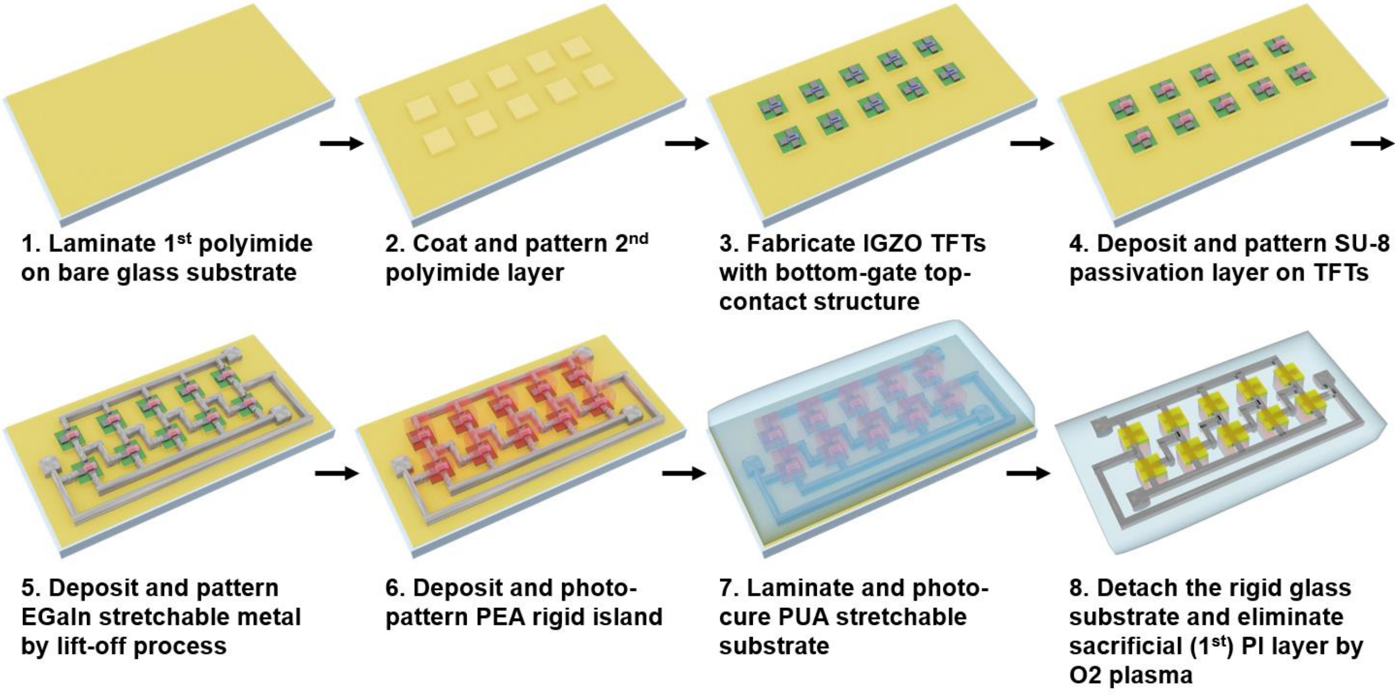

Supplementary Fig. 1 Schematic of the fabrication process flow.

First, a 3 μm-thick polyimide (PI) sacrificial layer is coated on a glass substrate. PI film has high mechanical durability and significant thermal resistance, ensuring good substrate compatibility during the fabrication process. Next, an additional 3 μm-thick PI layer is coated and photo-patterned to form the first rigid island. Then, amorphous indium-gallium-zinc-oxide (*a*-IGZO) transistors are fabricated using standard vacuum deposition and photolithography processes. Stretchable conductors (liquid metals) are deposited and patterned as interconnection by photolithography and lift-off methods (see **Supplementary Fig. 3**). Afterward, the *a*-IGZO transistors are passivated with SU-8 and a second rigid island is formed using PEA. Both SU-8 and PEA are deposited by spin coating and then patterned by photolithography. On the fabricated devices, PUA is spin-coated and photo-cured which served as the stretchable substrate. The whole devices are soaked in water and then physically detached from the glass substrate. Finally, the polyimide sacrificial layer on the back side of the device is removed using oxygen plasma to expose the electrode contacts.

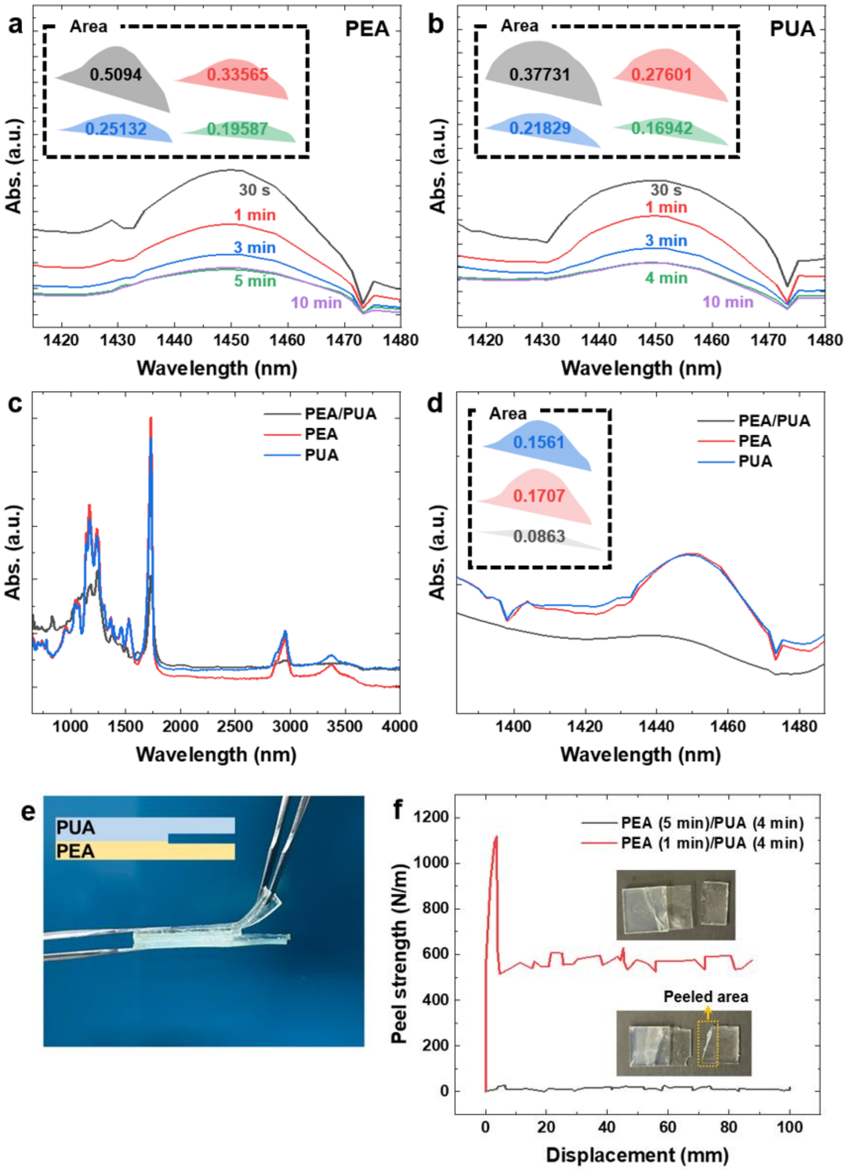

Supplementary Fig. 2 Chemical and mechanical characterization of PEA, PUA, and PEA/PUA composites. a-b, Integrated area analysis of the FTIR characteristic peak at 1450 cm^{-1}, indicative of the acrylate group's double bond, for **a,** PEA and **b,** PUA subjected to various UV-curing durations. **c,** Comprehensive FTIR spectra for PEA (5-min cure), PUA (4-min cure), and a combination of PEA (1-min cure)/PUA (4-min cure). **d,** Comparative analysis of the integrated area for the 1450 cm^{-1} peak, representing the double bond of the acrylate group, in fully cured PEA (5 min), PUA (4 min), and PEA (1 min)/PUA (4 min) samples. **e,** Photograph of the prepared PEA/PUA sample utilized in the peel test, comprising PEA cured for 1 min and PUA cured for 4 min. **f,** Graphical representation of peel strength versus displacement for samples with fully cured PEA (5 min)/PUA (4 min) and semi-cured PEA (1 min)/PUA (4 min). The adhesive interface between PUA and PEA in these tests measured 20 mm by 20 mm.

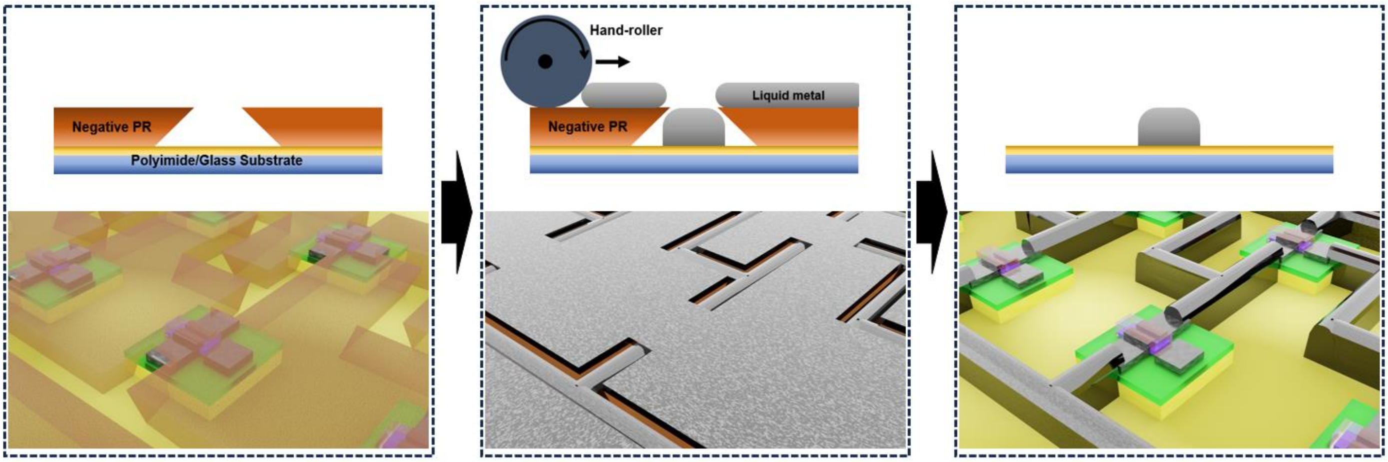

1. Spin-coat the negative photoresist and create the pattern with a deep undercut

2. Spread the liquid metal using a hand-roller

3. Pattern the liquid metal through lift-off process, which involves immersing it in acetone for 2 hours and gently spraying with acetone

Supplementary Fig. 3 Schematic showing the patterning process of EGaIn liquid metal. With the eutectic gallium indium evenly laminated on the patterned negative photoresist, the stretchable liquid metal interconnection is patterned by the lift-off process.

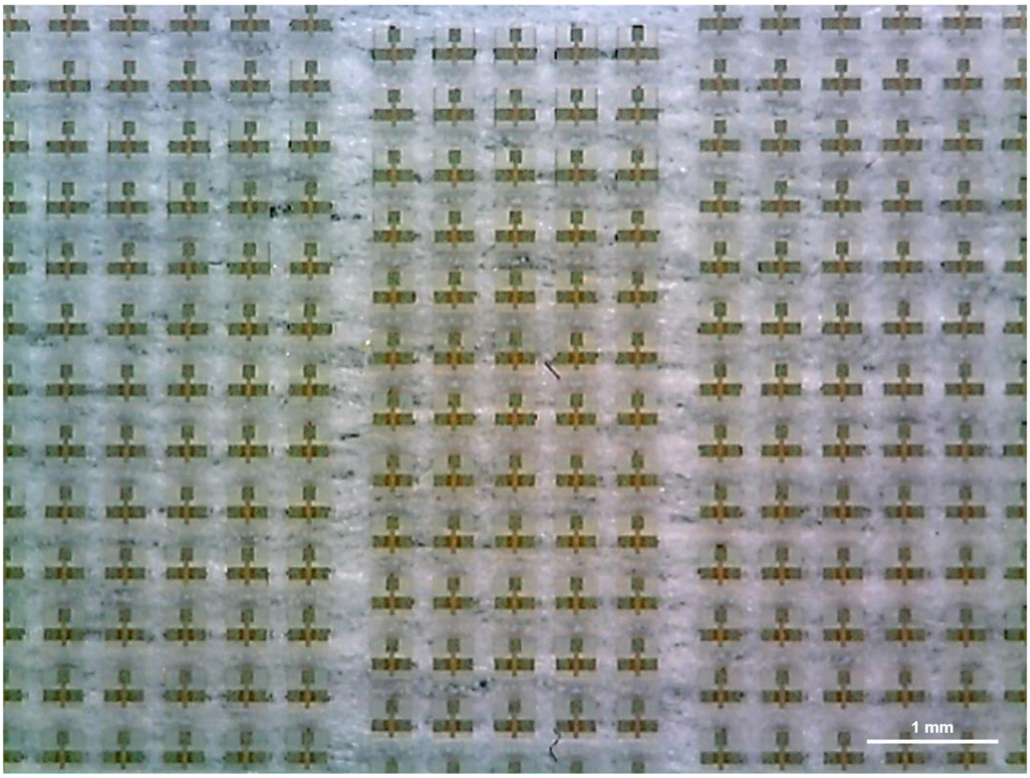

Supplementary Fig. 4 Densely arranged stretchable device array. Stretchable transistor array on the elastic substrate with a device density of 442 transistors/cm^2. (Scale bar, 1 mm)

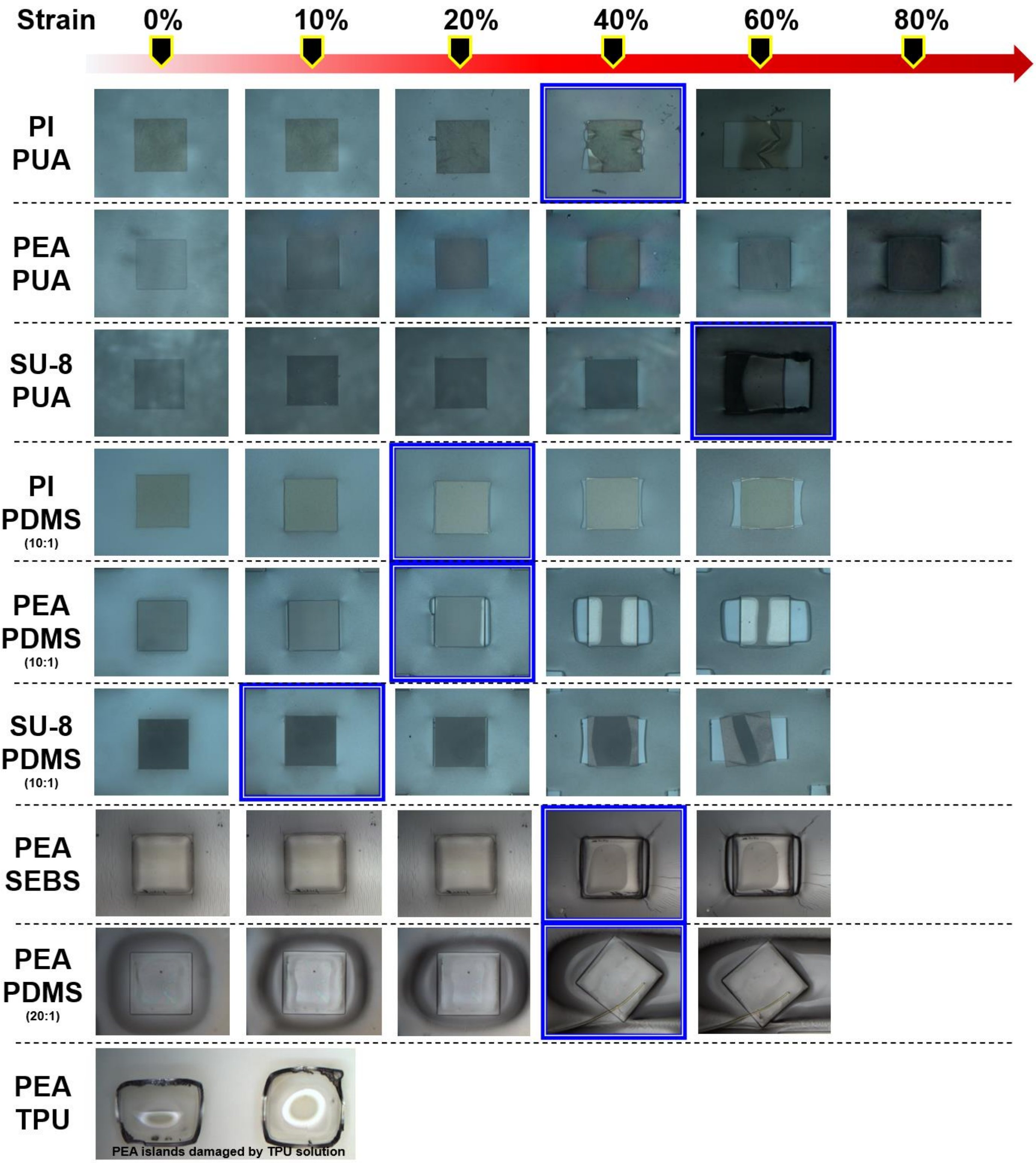

Supplementary Fig. 5 Delamination resistance dependent on materials of rigid island and elastomer. Optical microscopic image of 9 different combination of island (PI, PEA, SU-8) and elastomeric materials (PUA, PDMS, TPU, SEBS) under tensile strain. The time when delamination occurred on each substrate was indicated with blue border. Each square island is 350 μm × 350 μm.

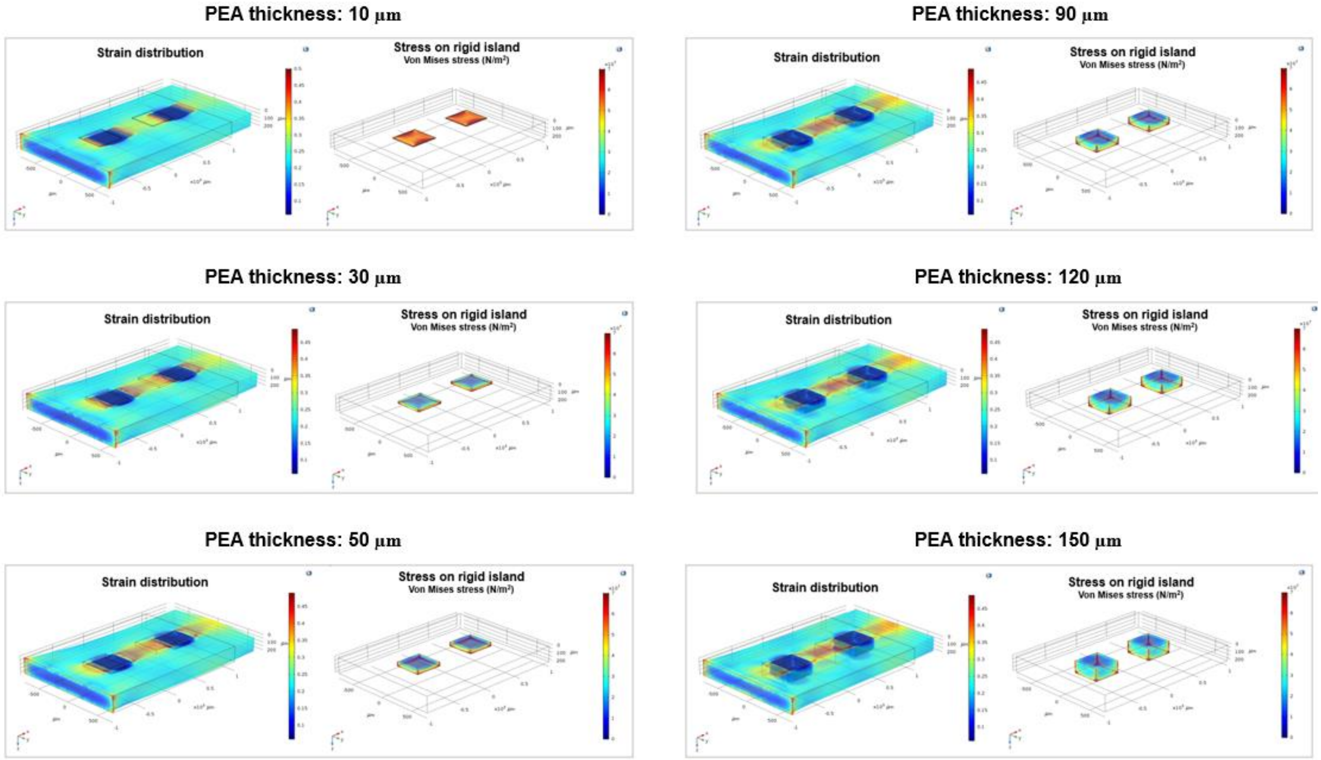

Supplementary Fig. 6 Strain and stress analysis according to rigid island thickness. Strain distribution on whole geometry and stress distribution on the rigid island under 30% of tensile strain with variation of the thickness of PEA rigid islands.

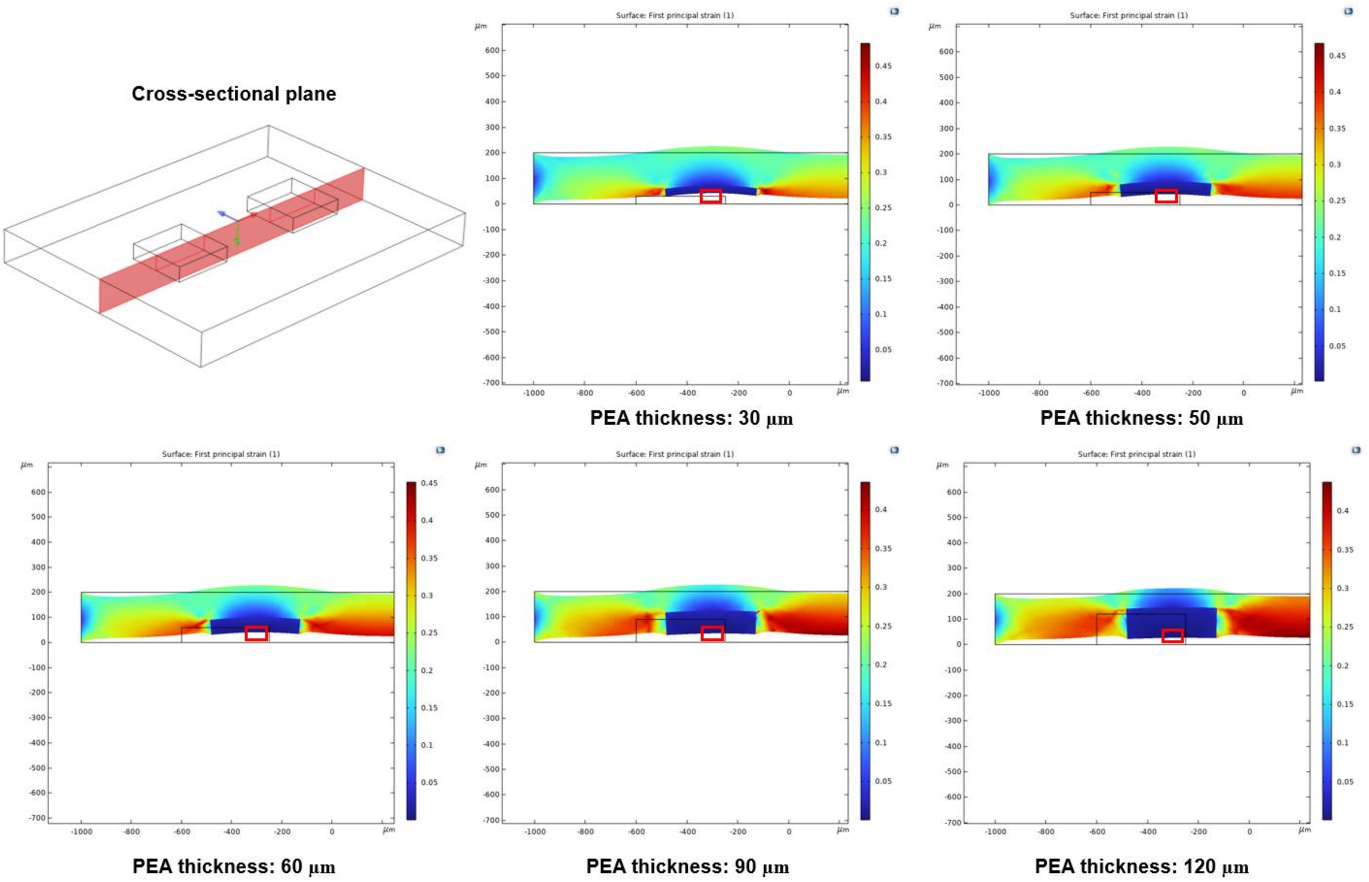

Supplementary Fig. 7 Cross-sectional image of strain distribution under 30% of tensile strain with variation of the thickness of PEA rigid islands.

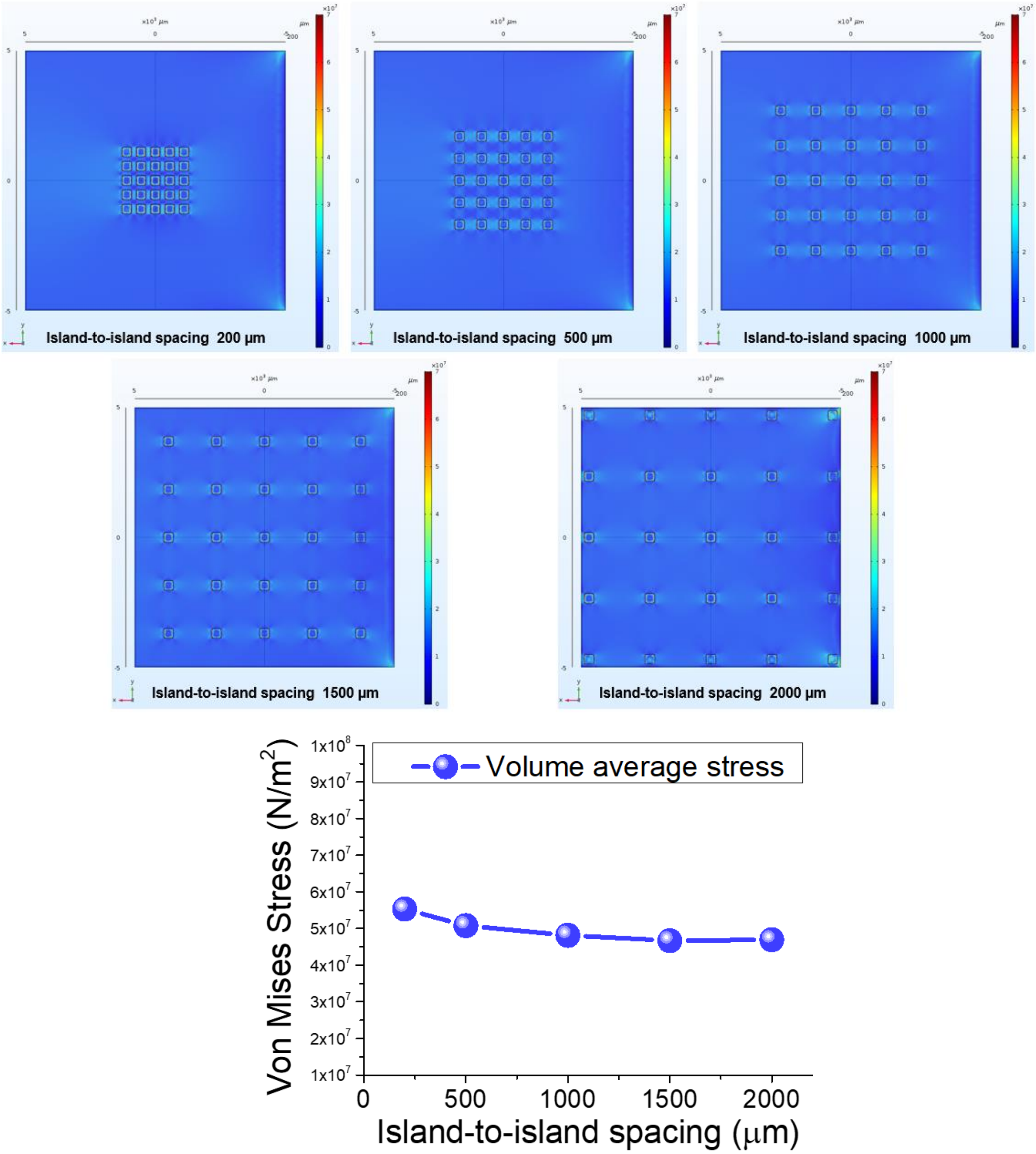

Supplementary Fig. 8 Stress distribution with the tensile strain of 30% for different island-to-island distance.

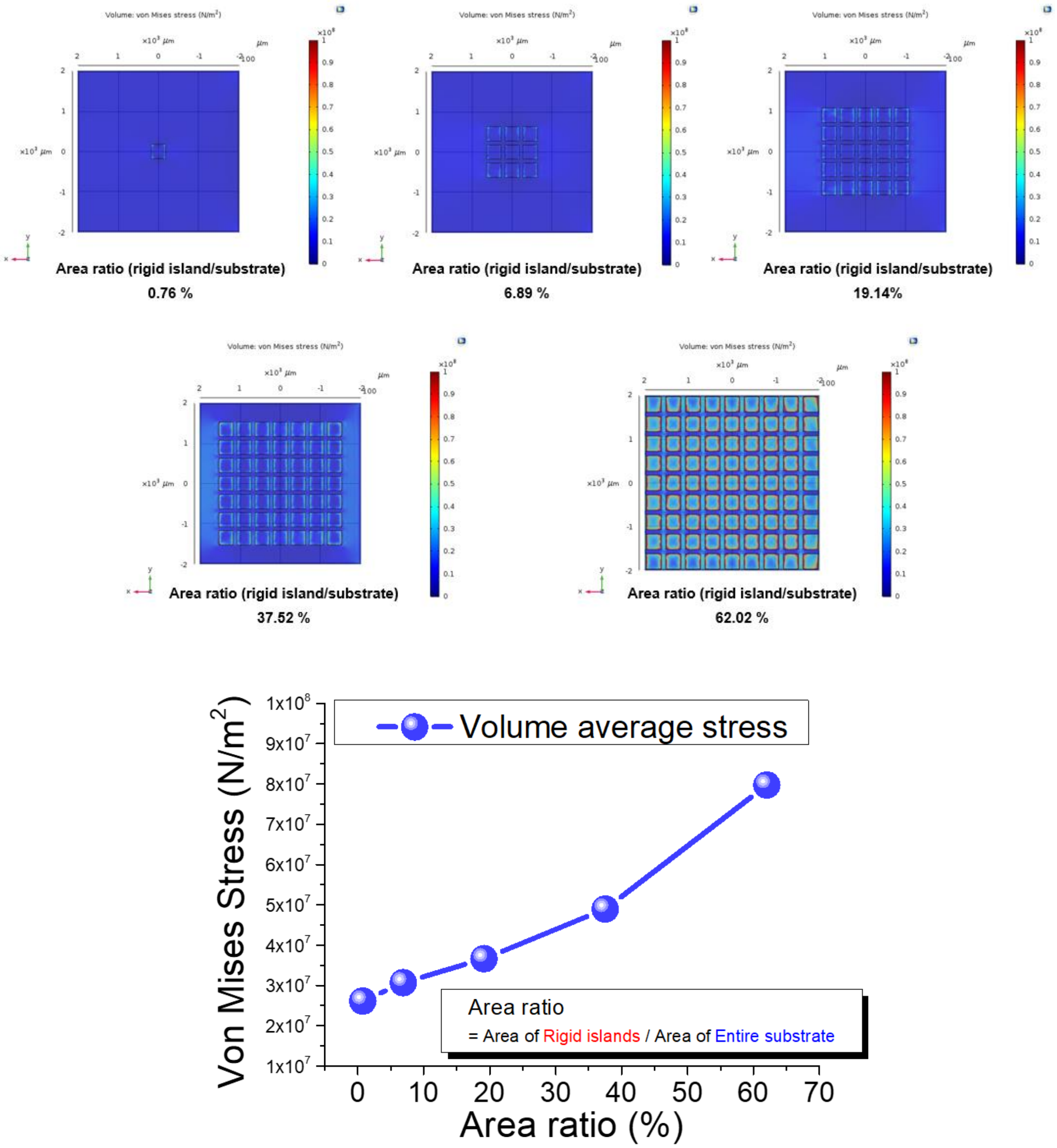

Supplementary Fig. 9 Stress distribution under the tensile strain of 30% as the density of the rigid islands varies.

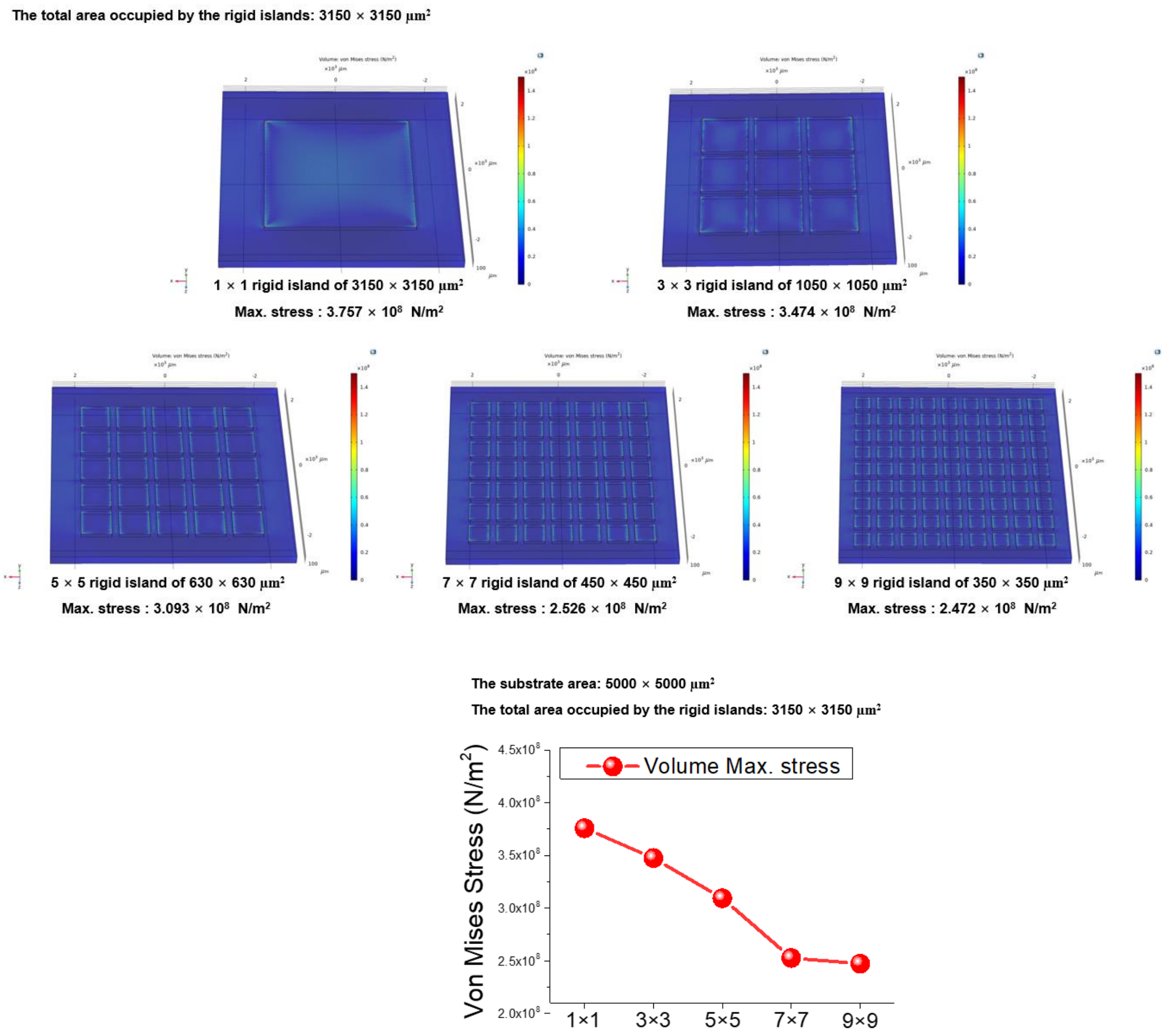

Supplementary Fig. 10 Stress distribution as the single rigid island size varies. Volume maximum stress under the tensile strain of 30% as the size of single rigid island varies while rigid island-covered portion is fixed.

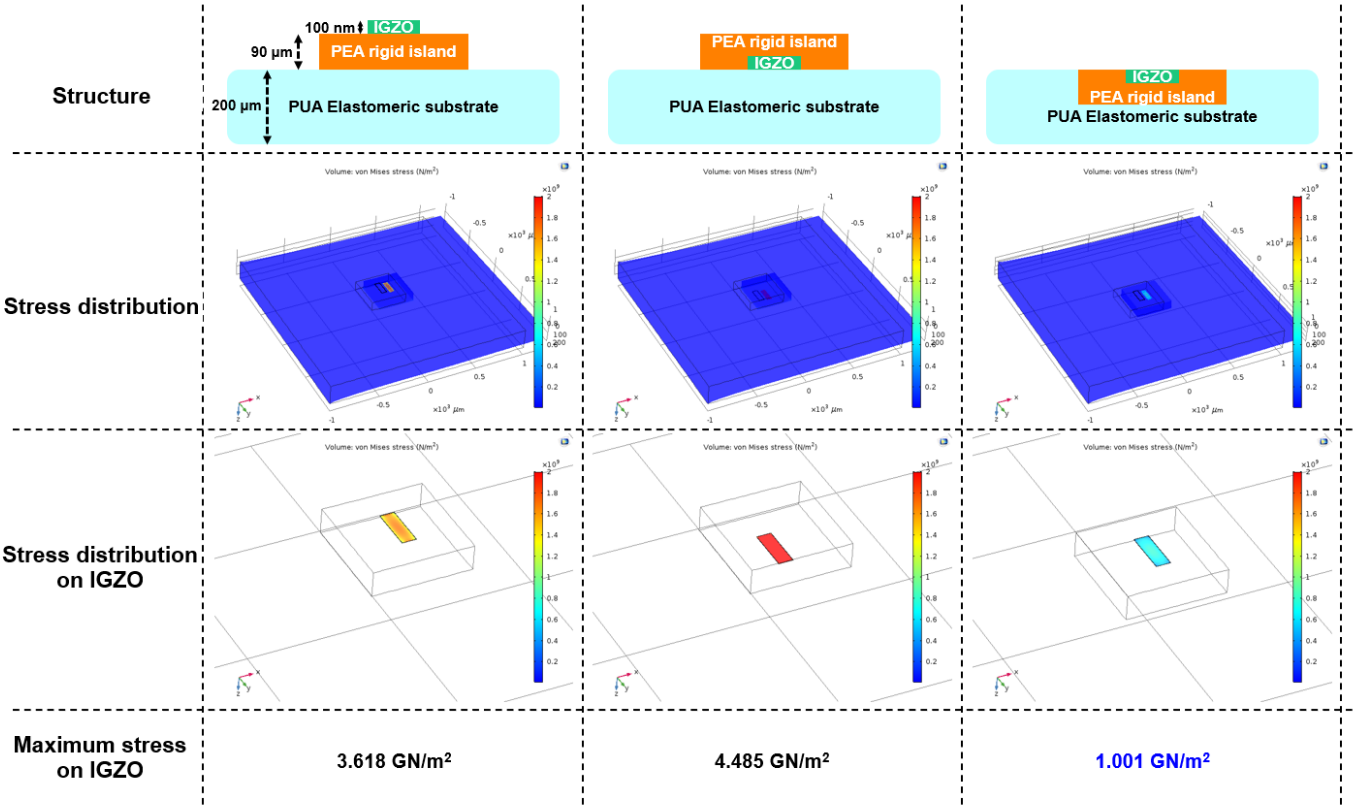

Supplementary Fig. 11 Stress distribution as the structure of rigid island and substrate varies. Stress distribution with the tensile strain of 30% for different architectures which utilize rigid island. The mechanical stress experienced by the IGZO metal-oxide layer in each architecture was analyzed when subjected to the applied tensile strain.

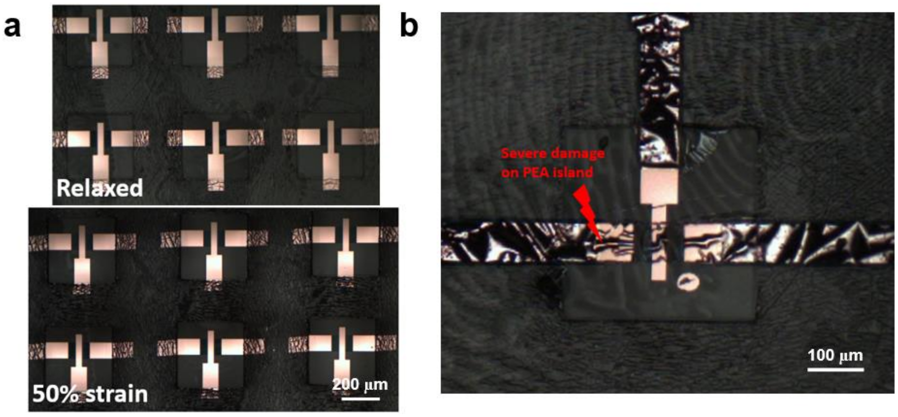

Supplementary Fig. 12 Mechanical damage on the devices within PEA/PUA substrate attributable to liquid metal wiring. a, Optical microscopic images of oxide transistors on PEA/PUA substrate. (Scale bar, 200 µm) **b,** Optical microscopic images of oxide transistors on PEA/PUA substrate interconnected through liquid metal wiring. (Scale bar, 100 µm) ff

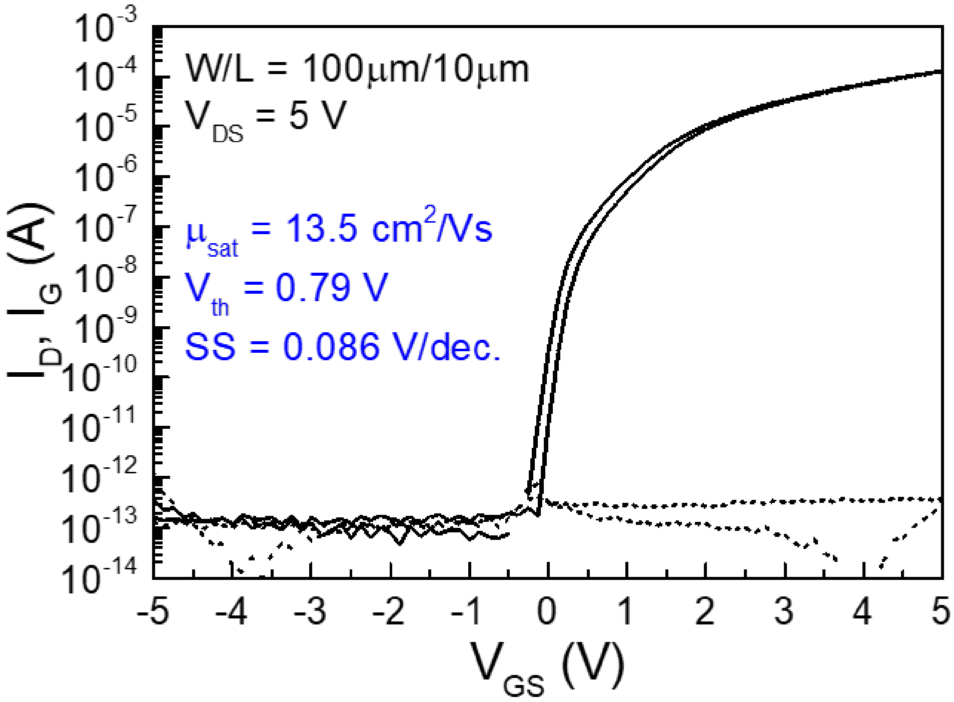

Supplementary Fig. 13 Transfer characteristics of *a*-IGZO TFT fabricated on the bare glass.

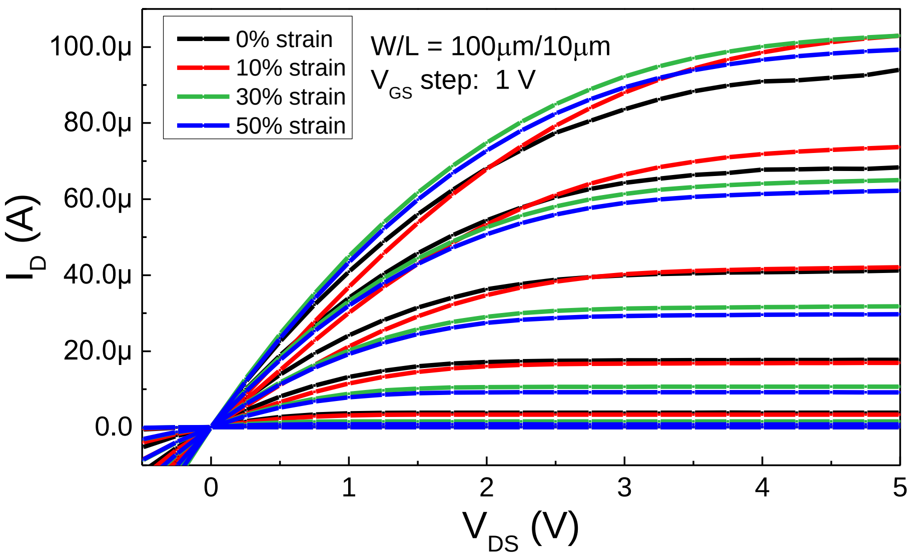

Supplementary Fig. 14 Output characteristics of stretchable *a*-IGZO TFT under tensile strain up to 50%.

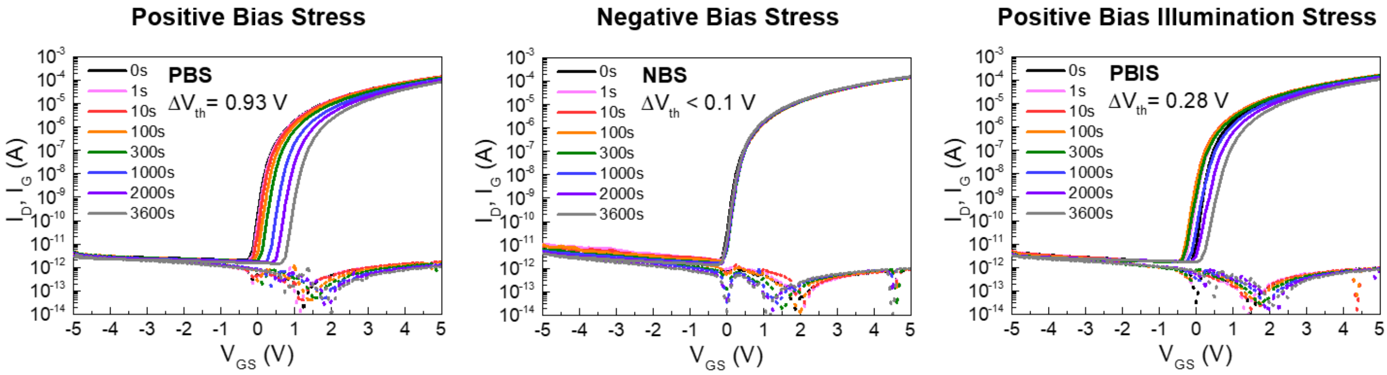

Supplementary Fig. 15 Bias stability tests for stretchable *a*-IGZO TFT. Positive bias stress (PBS), negative bias stress (NBS), and positive bias illumination stress (PBIS) tests for stretchable *a*-IGZO TFT. The conditions of bias and illumination stress are $V_{GS} = \pm 5$ V, $V_{DS} = 0.1$ V, light power = 3.65 mW/cm^2, and light wavelength = 320 nm to 1100 nm.

The *a*-IGZO TFTs exhibited a positive shift in threshold voltage under PBS conditions, with ΔV_{th} of +0.93 V. This phenomenon may be explained by the entrapment of electrons at the interface between the channel and gate dielectric, or the adsorption of oxygen molecules on the surface of the back channel[1,2]. The presence of the overlayered SU-8 and PEA rigid island layer reduces the impact of oxygen molecule adsorption at the back-channel region, accordingly they exhibited relatively small V_{th} shift. Under NBS, *a*-IGZO TFTs showed negligible difference of threshold voltage smaller than 0.1 V. In the case of PBIS, the negative shift occurred due to the influence of illumination up to 100 s, but then the effect by PBS was increased, and the threshold voltage was changed by 0.28 V at 3600 s.

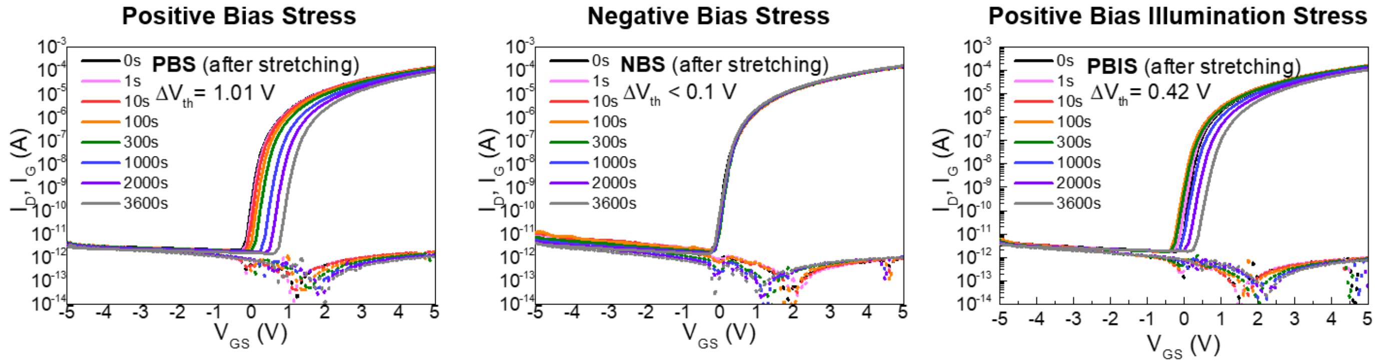

Supplementary Fig. 16 Bias stability tests after stretching. PBS, NBS, and PBIS of stretchable *a*-IGZO TFT under 30% of tensile strain.

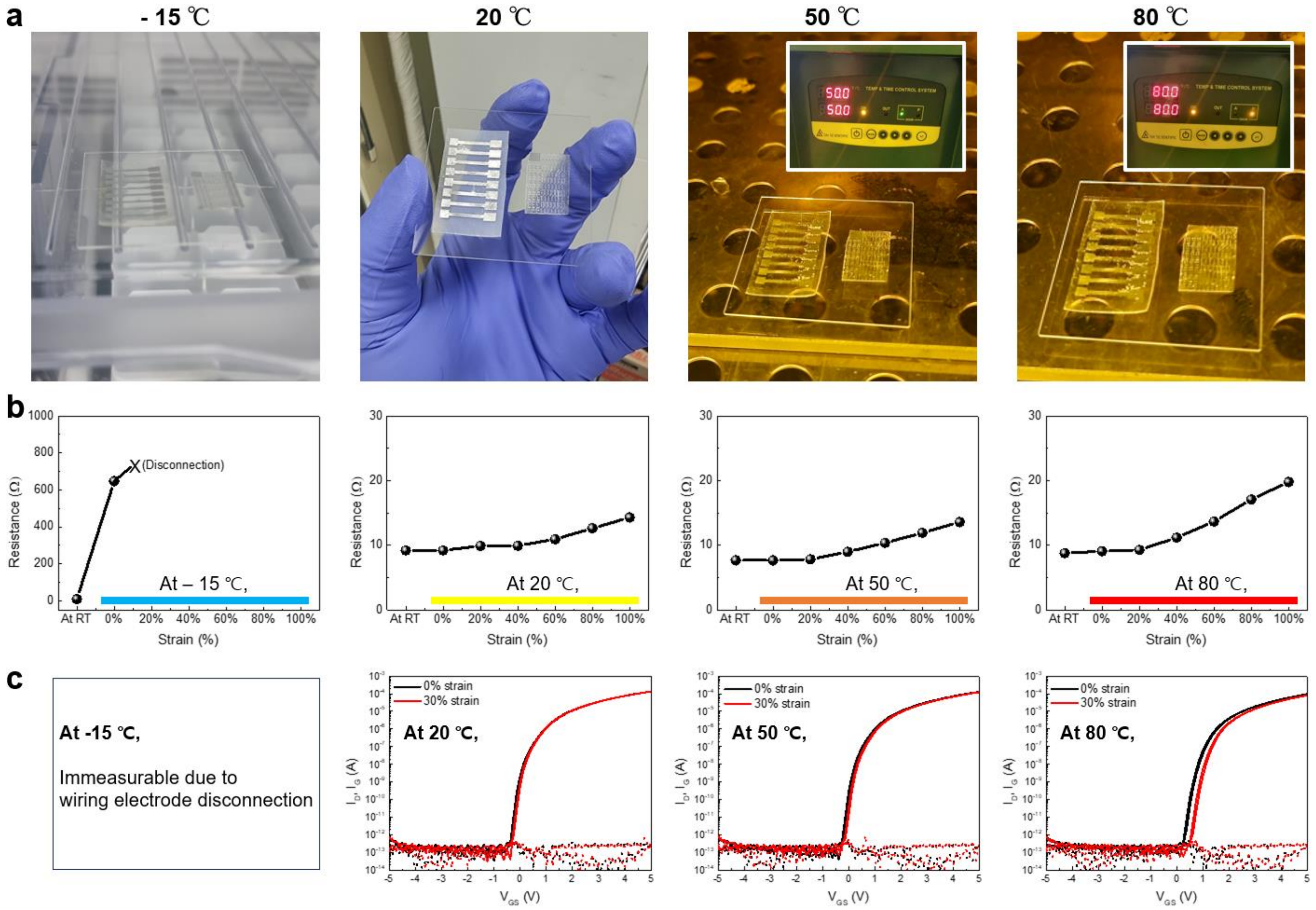

Supplementary Fig. 17 Temperature-dependent durability of liquid metal interconnects and transistors. **a,** Photographs of EGaIn liquid metal interconnects and transistors exposed to several temperature condition **b,** Electrical resistance variation of EGaIn liquid metal interconnects and **c,** transistor characteristics under tensile strain as the temperature varies.

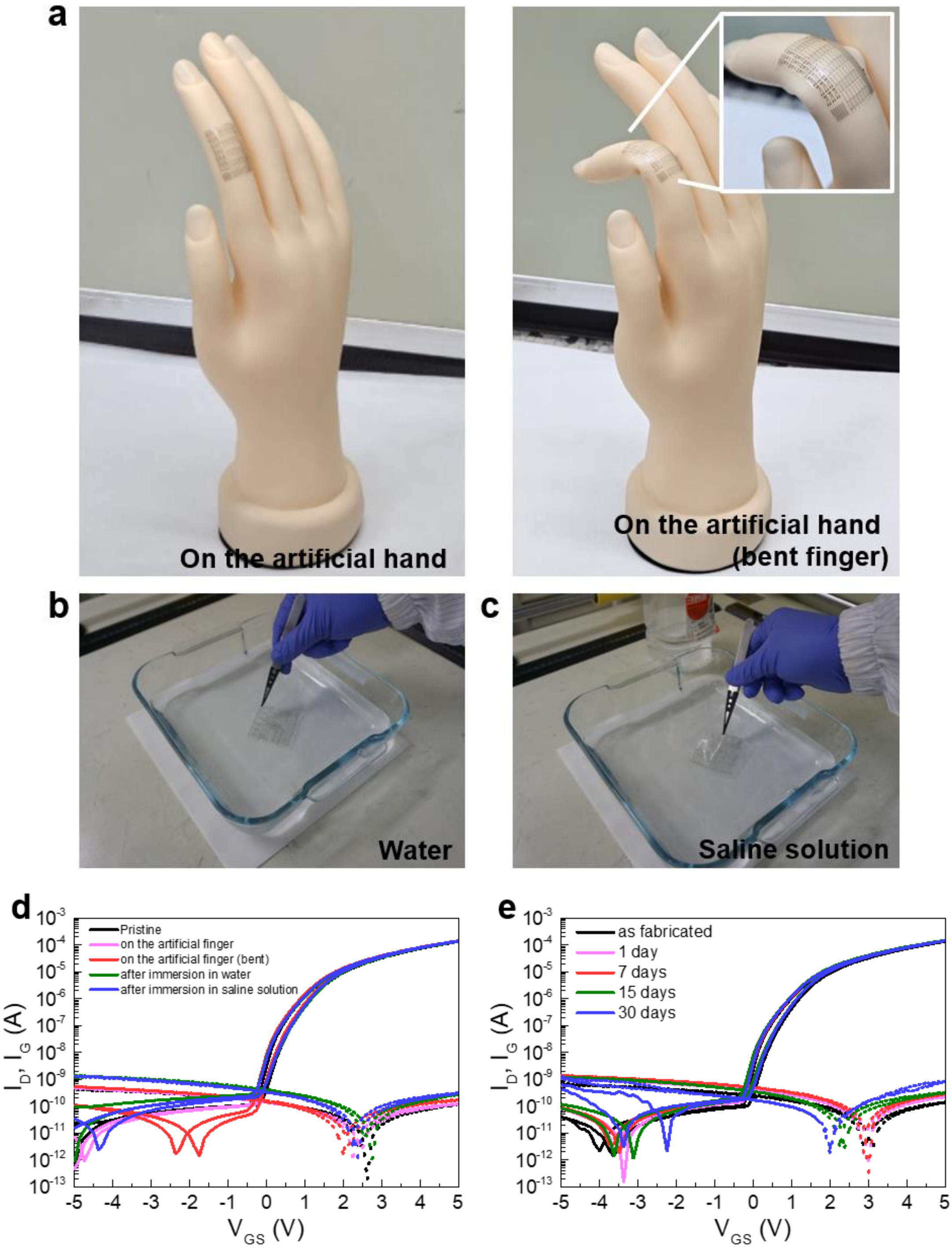

Supplementary Fig. 18 The durability tests of stretchable *a*-IGZO transistors under various environmental conditions. Stretchable *a*-IGZO devices **a,** attached to the finger of the artificial hand, **b,** immersed in water, and **c,** immersed in saline solution. **d,** Device characteristics after different environmental condition. **e,** Device characteristics over time in ambient atmosphere

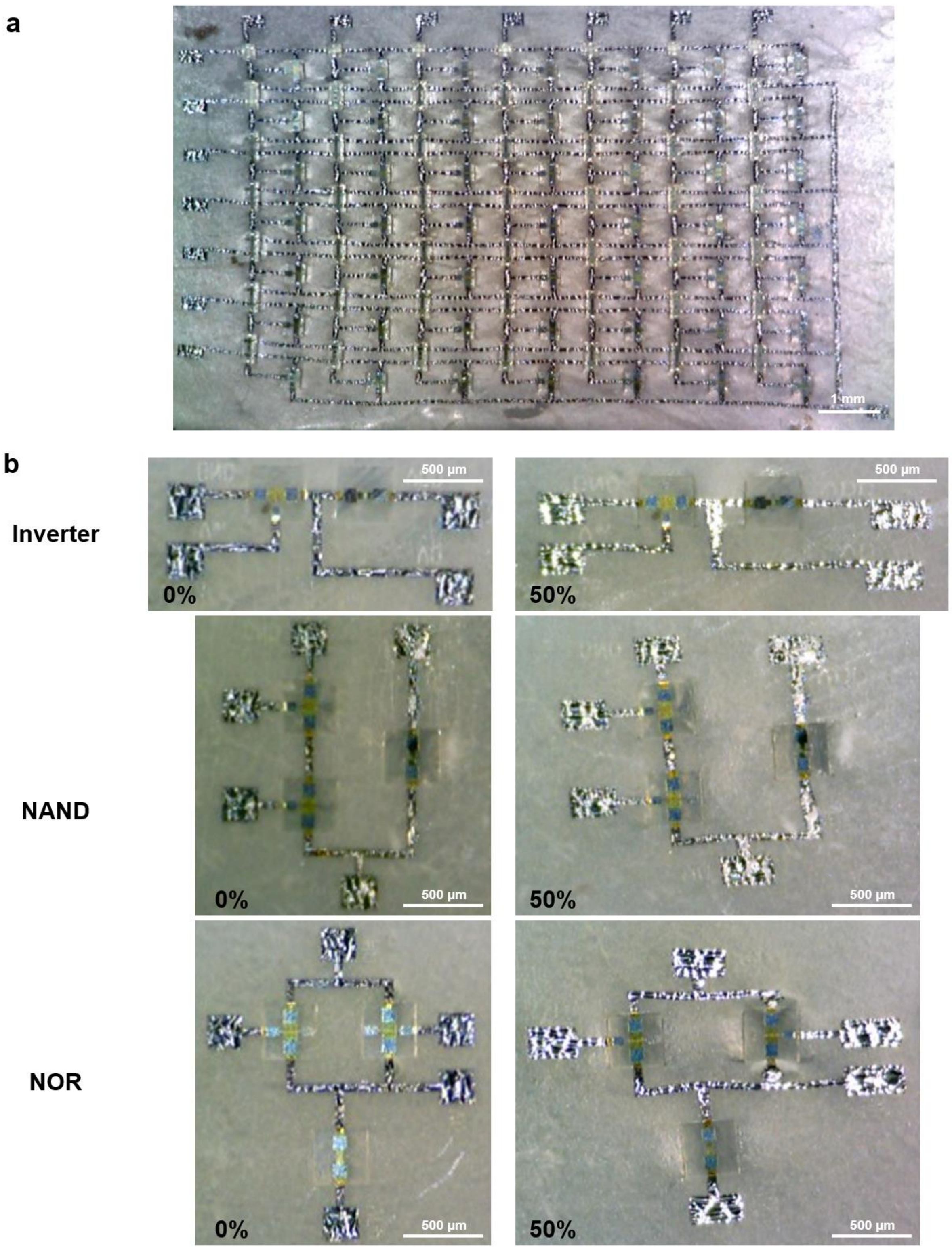

Supplementary Fig. 19 Stretchable circuits under harsh tensile strain. Micrographs of **a,** 7×7 stretchable transistor array and **b,** logic gates under 50% strain.

Material	Mobility	Structural designs	Max. Stretchability	Device density	Features	Published year
ZnO	1.15 cm^2/Vs	Wavy/Neutral plane	5 %	225 cm^{-2}	Single transistor	2010
IGZO	12.83 cm^2/Vs	Wrinkle formation, roll transfer	5 %	204 cm^{-2}	Single transistor	2013
IGZO	11.3 cm^2/Vs	Wavy	210 %	400 cm^{-2}	Common source amplifier<br>Rectifier	2015
IGZO	22 cm^2/Vs	Rigid island/Neutral plane	25 %	Not specified	Single transistor	2016
IGZO	13.7 cm^2/Vs	Buckled structure	5 %	1092 cm^{-2}	Inverter, NAND<br>Rectifier	2017
IGZO	1.2 cm^2/Vs	Mesa-shaped pillars	20%	36 cm^{-2}	inverter	2018
IGZO	1.5 cm^2/Vs	Rigid island<br>Liquid metal interconnect	40 %	4 cm^{-2}	4 × 4 active matrix	2018
IGZO	Not specified	Wavy	60 %	124 cm^{-2}	Single transistor	2019
IGZO	17.27 cm^2/Vs	Stiff stripes	50 %	144 cm^{-2}	Single transistor	2019
IGZO	14 cm^2/Vs	Rigid island/CNT buried layer	70 %	56 cm^{-2}	Single transistor	2019
IZO	2.24 cm^2/Vs	Rigid island<br>serpentine electrode	30 %	58 cm^{-2}	Strain sensor	2019
IGTO	25.4 cm^2/Vs	Rigid island	300 %	Not specified	Single transistor	2020
IGZO	13.2 cm^2/Vs	Rigid island	30 %	135 cm^{-2}	Inverter, NAND	2021
IGZO	24.9 cm^2/Vs	Multi-layered polymer island	30 %	1 cm^{-2}	Single transistor	2022
ITO	56.2 cm^2/Vs	Serpentine stiff string	100 %	30000 cm^{-2}	Inverter	2022
IGZO	12.7 cm^2/Vs	Molecular-tailored rigid island<br>Liquid metal interconnect	50 %	442 cm^{-2}	Inverter, NAND, NOR<br>7-stage ring oscillator<br>7 × 7 active matrix	This work

Supplementary Table 1 Comparison of stretchable metal oxide thin film transistors.

Sample	UV-curing durations	R^2 index	Related figure
PEA	30 s	0.9935	Supplementary Fig. 2a
	1 min	0.9906	
	3 min	0.9924	
	5, 10 min	0.9928	
PUA	30 s	0.9968	Supplementary Fig. 2b
	1 min	0.9953	
	3 min	0.9921	
	4, 10 min	0.9938	
PEA/PUA	PEA (1 min)/PUA (4 min)	0.9925	Supplementary Fig. 2d
PEA	4 min	0.9961	
PUA	5 min	0.9955	

Supplementary Table 2 The coefficient of determination (R^2) of FTIR spectral data. R^2 values that indicate the degree of agreement between the fitted graphs and the original FTIR spectra regarding **Supplementary Fig. 2**.

Layer	Material	Young’s modulus (GPa)	Density (kg•m^{-3})	Poisson’s ratio
PI rigid island	Polyimide	3.1	1300	0.34
PEA rigid island	PEA	1.2	1200	0.38
Elastic substrate	PUA	0.025	1100	0.46
S/D metal	Mo	315	10200	0.29
Active	IGZO	130	6500	0.36
Stretchable metal	EGaIn	10^{-8}	6280	0.45

Supplementary Table 3 The physical properties of the materials for finite element analysis. The material properties used for the COMSOL simulation were obtained from various sources, including measurement, existing literature and the COMSOL software itself. For EGaIn, which is liquid, it is not possible to define Young's modulus and Poisson's ratio. Therefore, within the operational range of the program, the values were set to be as close as possible to those of a liquid.

Supplementary References

1. Suresh, A. & Muth, J. F. Bias stress stability of indium gallium zinc oxide channel based transparent thin film transistors. *Appl Phys Lett* **92**, 033502 (2008).
2. Park, C.-Y. *et al.* High-performance ITO/a-IGZO heterostructure TFTs enabled by thickness-dependent carrier concentration and band alignment manipulation. *Ceram Int* **49**, 5905–5914 (2023).
